# How to evade a coevolving brood parasite: egg discrimination versus egg variability as host defences

**DOI:** 10.1098/rspb.2011.0401

**Published:** 2011-04-13

**Authors:** Claire N. Spottiswoode, Martin Stevens

**Affiliations:** 1 Department of Zoology, University of Cambridge, Downing Street, Cambridge CB2 3EJ, UK; 2 DST/NRF Centre of Excellence at the Percy FitzPatrick Institute, University of Cape Town, Rondebosch 7701, South Africa

**Keywords:** coevolution, egg colour, egg pattern, vision

## Abstract

Arms races between avian brood parasites and their hosts often result in parasitic mimicry of host eggs, to evade rejection. Once egg mimicry has evolved, host defences could escalate in two ways: (i) hosts could improve their level of egg discrimination; and (ii) negative frequency-dependent selection could generate increased variation in egg appearance (polymorphism) among individuals. Proficiency in one defence might reduce selection on the other, while a combination of the two should enable successful rejection of parasitic eggs. We compared three highly variable host species of the Afrotropical cuckoo finch *Anomalospiza imberbis*, using egg rejection experiments and modelling of avian colour and pattern vision. We show that each differed in their level of polymorphism, in the visual cues they used to reject foreign eggs, and in their degree of discrimination. The most polymorphic host had the crudest discrimination, whereas the least polymorphic was most discriminating. The third species, not currently parasitized, was intermediate for both defences. A model simulating parasitic laying and host rejection behaviour based on the field experiments showed that the two host strategies result in approximately the same fitness advantage to hosts. Thus, neither strategy is superior, but rather they reflect alternative potential evolutionary trajectories.

## 1. Introduction

Coevolutionary arms races between parasites and their hosts can be a significant driving force in evolution, and in avian brood parasites have led to substantial changes in phenotypic diversity and behaviour [1]. For example, many brood parasites have evolved highly mimetic eggs to evade detection by hosts [2], and manipulative begging calls to elicit increased parental care [3]. In response, hosts can defend themselves with a range of counter-adaptations, including nest defence against brood parasites [4,5], and rejection of foreign eggs [2,6] or chicks [7]. Much work investigating coevolution at the egg stage has focused primarily on the parasite's perspective, because selection for improved parasitic mimicry is clearly driven by rejection behaviour of hosts [2]. From the host's perspective, successful rejection of parasitic eggs is a function both of the host's level of egg discrimination, and of the difference in appearance between parasitic and host eggs [6,8]. Thus, when the arms race reaches a point where parasites have evolved mimetic eggs, hosts may have two major defence strategies at the egg stage [9]: first, they can improve their level of egg discrimination, by refining their ability or decision rules used to distinguish differences in egg appearance between parasitic eggs and their own. Second, they can shift their own egg phenotype away from that of the parasite and other hosts, rendering parasites easier to detect.

Much evidence for the first mechanism, elevated host discrimination, comes from the common cuckoo *Cuculus canorus*. It has a range of host-specific races (gentes) each with a different level of host mimicry [2], and those facing stronger host rejection show superior mimicry [2,10]. The second potential mechanism, shifting host phenotypes, has been modelled theoretically [11,12]. Hosts are expected to shift their phenotypes away from the original host and mimetic parasite phenotype, and to diversify egg appearance through frequency-dependent selection favouring the rare kind, rendering it harder for a parasitic female to match any one host clutch well enough to be accepted. Accordingly, scoring of egg appearance has revealed that host species of the common cuckoo that show higher levels of egg rejection also have subtly greater variation in appearance between clutches, and lower variation within them [13,14]. However, the common cuckoo and its hosts comprise a young system, with a relatively recent origin of currently observed gentes [15], implying comparatively short periods of coevolution between parasite and hosts. In contrast, in other systems among-clutch variation is much more phenotypically extreme (e.g. the hosts of certain African and Asian cuckoos, and the African cuckoo finch [16]), perhaps reflecting greater evolutionary age. In this paper, we refer to among-clutch variation as ‘polymorphism’, following previous studies of phenotypic diversity as a defence against parasites and predators (e.g. [16–18]); here, we are referring to extreme although continuous levels of intraspecific variation, rather than classical discontinuous polymorphisms (which are also classically known to be genetically based, cf. polyphenisms).

Two escalating defences (discrimination and polymorphism) can thus contribute to the successful rejection of parasitic eggs. While these defences are not necessarily mutually exclusive, proficiency in one should reduce selection on the other: the more successfully parasitic eggs are rejected, the weaker selection will be on either trait. Highly polymorphic species may be effective rejectors even with relatively crude egg discrimination, because parasites can rarely achieve a good match. Similarly, highly discriminating rejectors should receive fewer potential benefits from evolving among-clutch variation. It is therefore unlikely that both defences will be simultaneously maximized, especially as there may be costs associated with either strategy (see §4); rather, across host species, we would expect to find a mixture of both defences, the sum of which enables successful rejection of parasitic eggs. While polymorphism and discrimination are predicted to be inversely related to one another, their relative contributions should be determined by chance and species-specific constraints. These predictions can be investigated by comparing multiple host–parasite relationships, but this requires similar visual environments, consistent quantification of egg discrimination and variable polymorphism levels in multiple hosts.

**Figure 1.**
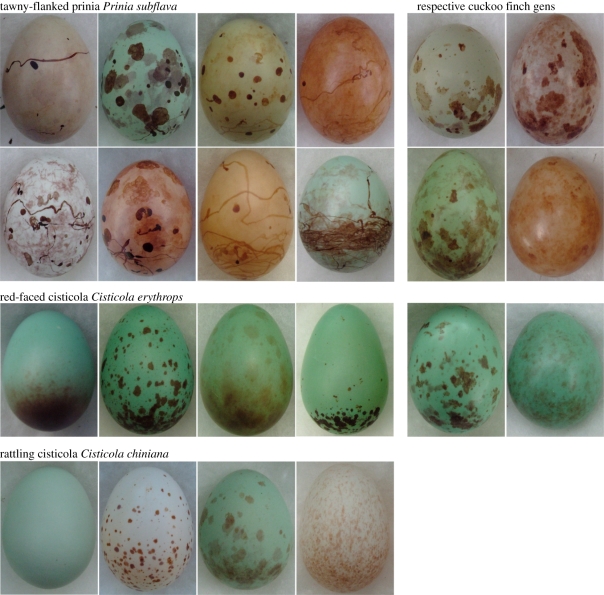
Representative host (left) and parasitic (right) eggs showing the range of polymorphism among females. Each egg came from a different clutch. No parasitic gens is shown for the rattling cisticola because it is not currently parasitized at our study site. (Note: not to scale.)

In this study, we experimentally investigated polymorphism and discrimination as two potential defence strategies in the hosts of an Afrotropical brood parasite, the cuckoo finch *Anomalospiza imberbis*. We investigate different evolutionary trajectories involving three co-occurring species: two currently exploited hosts and a third that shows strong egg discrimination behaviour, but is not currently parasitized at our study site. This system is particularly appropriate for this investigation owing to its evolutionary age (the cuckoo finch diverged from its closest relatives, the brood parasitic *Vidua* finches, *ca* 20 Ma [19]), and owing to the extreme levels of egg colour and pattern polymorphism shown by hosts and, correspondingly, by parasites (figure 1). Parasites lay their eggs haphazardly with respect to host morph, and incur high degrees of loss through host rejection [18]. Hosts are all sympatric at our study site in Zambia, and they build similar nest types in the same habitat, allowing their discrimination behaviour to be directly compared without potentially confounding variation in light conditions. We quantified egg colour and pattern in terms of current understanding of avian visual perception, which to our knowledge has not previously been applied to multiple evolutionary trajectories in brood parasites. This approach has two major advantages: first, it allows visual cues to be analysed in terms of how they relate to selection mediated by avian vision (as opposed to subjective assessment or by analysing shapes of reflectance spectra, neither of which necessarily relate to avian vision). Second, simultaneously quantifying multiple different aspects of pattern and colour allows us to identify precisely which visual cues are involved in egg discrimination and polymorphism, and their relative importance. These may differ among host species: we have previously found that one host species, the tawny-flanked prinia *Prinia subflava*, uses cues that reveal the most reliable information about egg identity [18]. Alternatively, particular egg features might be intrinsically better for establishing egg identity [20], or for visual discrimination tasks in general.

We first quantify overall levels of phenotypic variability among individuals (polymorphism) with respect to both egg colour and pattern in each host and its corresponding parasitic gens. Second, we compare host eggs with those of their corresponding parasitic gens to assess which traits differ most consistently between them (i.e. are on average least mimetic). Third, we use egg rejection experiments combined with visual modelling to identify which visual traits predict rejection decisions by different hosts (providing strong evidence that they are used as cues), and compare these to differences between real host and parasitic eggs. Fourth, using these experiments, we calculate an overall index of degree of discrimination that is comparable across species. Finally, for each host, we simulate the effectiveness of egg rejection in relation to real parasitic eggs of their corresponding gens, and thus compare the expected selective consequences of different defensive strategies (polymorphism versus discrimination). Overall, we predict that hosts with high levels of polymorphism will be less discriminating in absolute terms compared to hosts with lower levels of polymorphism, but that either strategy is an effective anti-parasite defence.

## 2. Methods

### (a) Study system

The cuckoo finch parasitizes a range of grass-dwelling warblers of the genera *Prinia* and *Cisticola*, and several well-defined host-specific races or ‘gentes’ specialize on different species (C. N. Spottiswoode & M. Stevens 2011, unpublished data). At our study site, it most commonly parasitizes the tawny-flanked prinia (rate of attempted parasitism greater than 19%) and also regularly parasitizes the red-faced cisticola *Cisticola erythrops* (rate of attempted parasitism greater than 8%). A third species, the rattling cisticola *Cisticola chiniana*, is common at this site but parasitism has never been recorded here (of 95 recent and 116 historical breeding records, the latter from 1969 to 1991; C. N. Spottiswoode & J. F. R. Colebrook-Robjent 2011, unpublished data); nonetheless, we cannot exclude the possibility of occasional parasitism attempts eliminated by host rejection before we detected them. Elsewhere in Africa, it has been recorded as a cuckoo finch host [21] and we provide evidence that it has been a host at our study site in the past. For brevity, we refer to all three warbler species as ‘hosts’. Examples of host and parasitic eggs are shown in figure 1.

We carried out fieldwork during January–March 2007–2009, within a *ca* 800 ha area on and around Musumanene Farm (16°47′ S, 26°54′ E) near Choma, southern Zambia. All hosts build woven nests with a side entrance, stitched among the broad leaves of small herbaceous shrubs (tawny-flanked prinias and red-faced cisticolas), or tucked into the base of a shrub or grass tussock (rattling cisticolas). Hosts pay strong fitness costs of parasitism since cuckoo finch hatchlings usually outcompete all host young [22]. Hosts removed foreign eggs by puncturing then ejecting them.

### (b) Field experiments

Detailed methods for field experiments are given in Spottiswoode & Stevens [18]. Briefly, we used conspecific eggs from other nests to experimentally parasitize hosts, then modelled potential predictors of egg rejection (from a candidate set of eight colour, luminance and pattern traits, detailed below). The potential for phenotypic mismatch in our experimental nests differed among host species since degree of polymorphism varied among them; however, this does not confound our estimate of the degree of discrimination (below) since we sought to present all hosts with more difficult rejection decisions than would be generated by randomly placing host eggs (see also [18]). We mimicked cuckoo finch laying behaviour by removing one host egg when we placed an experimental egg in a nest. A different host female was used for each experimental trial. All eggs were photographed in RAW format alongside a 17 per cent neutral grey card (Kodak) using a Fuji Finepix S7000 digital camera, and these photographs were used to quantify pattern (below). Reflectance spectra (for colour and luminance analysis) of the removed host egg were subsequently measured indoors (below), and the colour of the removed egg was taken as representative of the host clutch; this was justified by intermediate to high intra-class correlation coefficients (ICC or repeatabilities) of colour channel (CC) values (defined below) among eggs within a clutch (electronic supplementary material, table S1). Experimental nests were visited daily when possible and eggs were considered accepted if they remained for 3 days in the nest; experimental eggs that disappeared while the rest of the clutch remained in the nest were considered rejected.

### (c) Quantifying colour and pattern attributes

We measured reflectance spectra using an Ocean Optics USB2000 spectrophotometer, with a PX-2 pulsed xenon light source and an R400-7-UV/VIS reflectance probe (all Ocean Optics Inc., Dunedin, FL, USA), and with reference to a Spectralon 99% white reflectance standard (Labsphere, Congleton, UK). A slanted plastic sleeve held each egg at a constant distance (5 mm) and angle (45°) from the probe tip. Five measurements were taken from the egg's background colour (i.e. avoiding overlaid darker patterns) and the mean analysed. Irradiance (‘ambient light’) within nests was measured for each species in the field using a cosine-corrected probe (details in [18]). We then calculated the predicted photon catches of a bird's single and double cones [23], using sensitivity data of the blue tit *Cyanistes caeruleus* because its visual system is better studied than other bird species [24]; data are unavailable for our study species. Repeating the modelling with other higher passerine bird species' sensitivity made negligible difference to the results [18]. Double cone catch data were taken to indicate luminance, as achromatic information in birds seems to be provided by the double cones [25]. For colour, plotting the standardized single cone catch data (using relative cone catches to remove variations in absolute brightness) in avian tetrahedral colour space [23] indicated that all host species and parasite gentes were distributed along the same single plane in the colour space. This was confirmed by a principal component analysis (PCA) on a covariance matrix of these standardized single cone data: two principal components (PCs) explained 99.6 per cent of the variation in egg appearance (of which PC1 corresponded to 73.8%). We used this PCA as a basis to make an informed decision about how to encode colour in a biologically meaningful way based on the principle of opponent CCs, whereby opposing colours are encoded in antagonistic neural pathways (similar to the ‘red-green’ and ‘blue-yellow’ CCs in human vision; [25]) based on Komdeur *et al*. [26]. We treated each CC as a ratio, expressed as
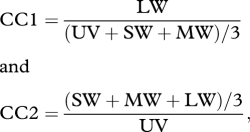
where LW, MW, SW and UV indicate longwave, mediumwave, shortwave and ultraviolet cone catches, respectively. Much of our egg colour analysis is based on these two opponent-style CC calculations (yielding CC1 and CC2).

To quantify pattern, we used an approach recently developed to quantify camouflage in cuttlefish [27] and mimicry in the common cuckoo [10]. Briefly, we used a ‘granularity’ method that decomposes calibrated digital images [28] of a pattern into the relative contribution of markings from different spatial scales (frequencies). We first linearized and calibrated the images of the eggs into reflectance data [28], extracted the MW channel (approximating to brightness [10]) and used Fourier transformation and bandpass filtering to generate a series of seven images capturing information at different spatial frequencies. Calculating the ‘energy’ (the sum of the squared pixel values [10,27]) of each of these seven images allows three measures of pattern to be derived: (i) the image with the maximum energy corresponds to the filter size that captured the most information, and reveals which marking size is most prevalent (‘filter size’, an inverse measure of marking size); (ii) the proportion of the total energy across all images contained by the filtered image with the highest energy (‘proportion energy’) measures how much the main marking dominates the overall egg pattern, with higher values indicating that one marking size dominates; and (iii) the total energy (‘total energy’) contained across all images, which is a measure of contrast between egg patterns and the background colour [10,27]. Additionally, we obtained two further measures of pattern (outlined in [10]) by thresholding the images into a binary format and calculating how much of the eggs were covered with markings; (iv) ‘proportion coverage’ is the average proportion of the egg's surface covered by markings rather than the background colour; and (v) ‘dispersion’ is the difference in pattern coverage between the narrow wide regions of the egg (details in [10] and [18]). All colour and pattern analyses were undertaken with custom-written programs in Matlab (Mathworks).

### (d) Quantifying levels of polymorphism

To investigate phenotypic variability and egg discrimination among host species, we devised a single measure of phenotypic distance between any two eggs in the population sample. This was defined as the Euclidean distance between two points in 10-dimensional space, defined by four colour variables (standardized single cone catch values), luminance information (double cone values) and the five pattern variables (dispersion, pattern proportion, filter size, proportion energy and total energy, as defined above). To make the scale comparable, each of the 10 variables was standardized by expressing it as a proportion of its maximum value across all groups. To estimate the overall phenotypic space occupied by each host or gens, we calculated the distance between every egg and every other egg in the sample, generating a matrix of distances. The grand mean across all eggs is an index of the overall degree of phenotypic variability in the population (‘multi-dimensional phenotypic space’; MDP space).

### (e) Quantifying levels of discrimination

We used the MDP distance across all colour, luminance and pattern attributes as a conservative measure of absolute phenotypic difference between host and experimental eggs. We considered all traits because we cannot infer from our egg rejection experiments which traits might historically have been under selection. We used the lower 25 per cent quartile value of rejected eggs as an index of the smallest phenotypic difference that was detectable to hosts; we used a quartile rather than a minimum value because the latter could be unduly influenced by outliers (e.g. hosts that had just seen an adult parasite), although conclusions would be unchanged whichever measure was used. High values imply that a foreign egg must be more different for it to be rejected by the host; i.e. that the host shows cruder discrimination (or greater ‘tolerance’ of dissimilar eggs [6]).

### (f) Statistical analyses

Sample sizes between different hosts and gentes differed, which could affect the outcome of the MDP space calculations. Therefore, for all analyses, we resampled the larger sample down to the sample size of the smallest: in each case, we drew a random subset of eggs from the population 999 times and carried out MDP space calculations on each. Statistical analyses were carried out using R [29]. For bivariate comparisons of egg traits (e.g. between host and parasite), we used two-sample *t*-tests for unequal variances (Welch's *t*-test) on ranked data, as recommend by Ruxton [30]. To model predictors of egg rejection, we compared logistic regression models (including first-order interactions) by constructing generalized linear models with binomial error structure. Final model selection was complicated by multiple competing models with similar AIC values, so we used an information theoretical approach to average a model set defined by *Δ*AIC less than 2 [31], using the R package MuMin. We first standardized predictors to have a mean of 0 and a s.d. of 1 such that model-averaged coefficients could be interpreted as standardized effect sizes [31].

### (g) Simulating selection on parasitic eggs

We used our logistic models of conspecific egg rejection experiments to estimate the proportion of real parasitic eggs likely to be rejected by each host species. We simulated a randomly laying cuckoo finch female by pairing randomly sampled host and cuckoo finch eggs (of the corresponding gens) 999 times, and for each pair calculating the phenotypic difference between them for each egg trait. Because for red-faced cisticolas the available sample of real cuckoo finch eggs was small (*n* = 7), to minimize the risk of identical host–parasite combinations, we chose 50 random host eggs in random order and compared each with a randomly chosen parasitic egg (thus ensuring a different comparison each time), and repeated this process five times, thus generating 250 host–parasite comparisons. We then substituted these standardized values into the rejection models (reported in the electronic supplementary material, table S3) to estimate the probability of egg rejection for each simulated laying event.

## 3. Results

### (a) How polymorphic are different hosts' and gentes' eggs?

**Figure 2.**
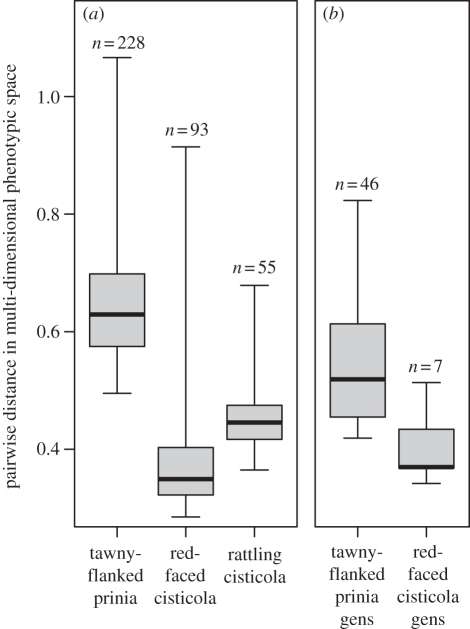
Degrees of polymorphism in egg phenotype among hosts and their parasites; whiskers indicate ranges. Values on *y*-axis refer to pairwise distances in 10-dimensional space (defined by four colour, one luminance and five pattern variables) between every egg and every other egg in the sample. Differences among (*a*) hosts and (*b*) gentes were unaffected by sample size, as shown by resampling. No data are shown for a rattling cisticola gens because this species is not parasitized at our study site.

In all species, there was relatively little phenotypic variation within clutches (electronic supplementary material, table S1); however, species differed in the level of among-clutch variation, i.e. polymorphism. We calculated MDP space for each host species and their respective cuckoo finch gens (figure 2). Tawny-flanked prinias laid the most polymorphic eggs (figure 2*a*; mean ± s.e. = 0.651 ± 0.007) and red-faced cisticolas the least (0.381 ± 0.010); rattling cisticolas (not currently a host) were intermediate (0.461 ± 0.013). These differences were highly significant for both the raw (Kruskal–Wallis test, 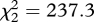, *p* < 0.001) and the resampled data (*p* < 0.001 for 100% of resamples with *n* = 55 per group; resampled mean ± s.e. for prinias = 0.651 ± 0.002 and for red-faced cisticolas = 0.382 ± 0.002). Correspondingly, cuckoo finch eggs laid in tawny-flanked prinia nests were more polymorphic (mean ± s.e. = 0.547 ± 0.015) than those laid in red-faced cisticola nests (0.404 ± 0.038) (figure 1*b*; *t*′ = −4.10, d.f. = 9.04, *p* = 0.003 on raw data; *p* < 0.05 for 40.2% of resamples with *n* = 7 per group; resampled mean ± s.e. for prinia gens = 0.547 ± 0.002).

Within each species, the eight individual aspects of egg appearance showed low levels of correlation with one another (*r*^2^ values; tawny-flanked prinias: range 0.0007–0.446, median = 0.019; red-faced cisticolas: range = 0.0001–0.285, median = 0.011; rattling cisticolas: range = 0.0001–0.277, median = 0.040); the only pair of traits that explained more than 30 per cent of the variance in one another were luminance (double cone catch) and CC1 values for tawny-flanked prinias.

### (b) Which egg traits do parasites mimic best?

For each of the two currently parasitized host species, we compared the degree of matching in each visual trait (electronic supplementary material, table S2). A similar test for pattern traits in tawny-flanked prinias alone has previously been published in Spottiswoode & Stevens [18]; it is reported again here for comparison, with a slightly larger sample size owing to the extraction of additional pattern data for non-experimental nests. For tawny-flanked prinias, we found that parasitic eggs were significantly different from host eggs with respect to: (i) one axis of colour (CC2: the level of UV stimulation compared with the other three cone types was higher in parasitic eggs than hosts'), (ii) dispersion (parasitic egg markings were more concentrated at one pole of the egg), (iii) proportion energy (parasitic egg markings were more dominated by a single marking size), and (iv) filter size (parasitic egg markings were generally larger). For red-faced cisticolas, we found that parasitic eggs differed from host eggs with respect to: (i) the other axis of colour (CC1: the level of LW cone stimulation compared with the other three cones was higher in parasitic eggs than hosts'), (ii) luminance (parasitic eggs were lighter), (iii) dispersion (parasitic egg markings were more evenly distributed across the egg), (iv) filter size (parasitic egg markings were smaller), and (v) total energy (parasitic egg markings were more contrasting).

### (c) Which traits do hosts use in rejecting foreign eggs?

Of 125 rejection trials in tawny-flanked prinias, 63 eggs were rejected and 62 accepted. Of 59 trials in red-faced cisticolas, 26 eggs were accepted and 33 rejected. Of 37 trials in rattling cisticolas, 14 eggs were accepted and 23 rejected. The data for tawny-flanked prinias have previously been published [18] and are reported again here for comparison; note, however, that the current paper uses colour differences between host and experimental eggs based on ‘opponent-style’ channels (see §2), rather than explicit perceptual distances as previously. This is because here we required a measure of each individual egg's colour, as well as differences between eggs.

Model-averaged linear models showing predictors of rejection are reported in electronic supplementary material, table S3. Both colour and pattern cues predicted rejection behaviour in tawny-flanked prinias, with the two together accounting for 26.7 per cent of the variance in rejection behaviour. Of this explained variance, colour accounted for 38 per cent, and three pattern traits (dispersion, proportion energy and filter size) together accounted for the rest. In red-faced cisticolas, we were able to explain a similar proportion (25.7%) of the variance in rejection behaviour. This was accounted for an interaction between one CC (CC1) and total energy (pattern contrast). We partitioned the data to examine how this interaction arose (electronic supplementary material, figure S2), and found that when the difference in total energy was low, hosts did not use colour to discriminate between accepted and rejected eggs (suggesting that an unmeasured additional variable must have accounted for rejected eggs that differed little in total energy); when the difference in total energy (i.e. difference in pattern contrast) was high, hosts rejected eggs with a large difference in colour but accepted eggs with a small difference in colour. In rattling cisticolas, 44 per cent of the variance in rejection behaviour was accounted for by two traits together, namely colour and filter size, with the latter contributing about two-thirds of the explained variation.

### (d) How discriminating are different host species?

The lower 25 per cent quartile value of phenotypic distance between host eggs and rejected experimental eggs reflects the smallest phenotypic difference that triggered host rejection; these values were 0.467 for tawny-flanked prinias, 0.283 for red-faced cisticolas and 0.374 for rattling cisticolas (electronic supplementary material, figure S1). This may be compared with a mean phenotypic distance between host eggs and real parasitic eggs of their corresponding gens (random pairings simulated following §2*g*; low values correspond to high average mimicry) of 0.625 ± 0.007 (range 0.096–1.516) for tawny-flanked prinias and 0.471 ± 0.017 (range 0.163–1.014) for red-faced cisticolas; these differed significantly (*t*′ = −10.92, d.f. = 407.4, *p* < 0.001).

### (e) How effective is current egg discrimination against parasites?

**Figure 3.**
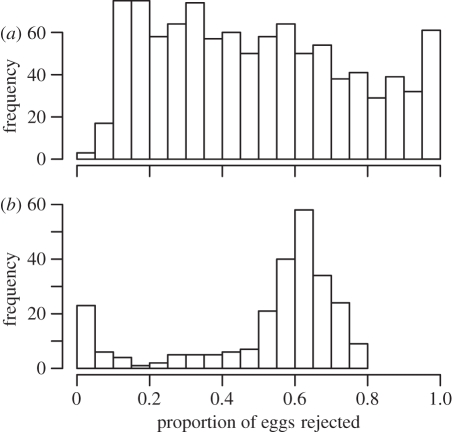
Rejection frequency by each host species of real cuckoo finch eggs laid by their respective parasitic gens, based on simulations using logistic models of egg discrimination experiments (see §2). Total frequencies add up to in (*a*) 999, tawny-flanked prinias, but in (*b*) 250, red-faced cisticolas (see §2).

Using simulations based on the egg rejection models derived from experimental data, we found that prinia–cuckoo finches laying randomly in tawny-flanked prinia nests have an average rejection probability of 0.492 ± 0.008 (range 0.047–0.999), and that the distribution of rejection probabilities was relatively even (figure 3*a*). Red-faced cisticola–cuckoo finches laying randomly in red-faced cisticola nests have an average rejection probability of 0.521 ± 0.014 (range 0.001–0.795); thus, very similar on average to prinias, but with a less even distribution (figure 3*b*).

## 4. Discussion

In this study, we have quantified polymorphisms in egg appearance and levels of egg discrimination in three sympatric species interacting with the same parasite, the cuckoo finch. Each species differed with respect to its level of egg polymorphism, its degree of discrimination in rejecting foreign eggs and which visual cues it used to do so. Refined egg discrimination and elevated egg polymorphism are two potential host counter-adaptations to escape a mimetic parasite. Consistent with the hypothesis that proficiency in one defence strategy should reduce selection on the other, we found that the species with the highest degree of polymorphism showed the crudest discrimination behaviour (tawny-flanked prinia). By contrast, the species with the least extravagant polymorphism was the most discriminating (red-faced cisticola). Correspondingly, red-faced cisticolas were parasitized by a more closely mimetic cuckoo finch gens than tawny-flanked prinias. A third species, the rattling cisticola, is not currently parasitized at our study site and was intermediate with respect to both polymorphism and discrimination. Predictive models testing the likelihood of host rejection of real parasitic eggs (based on egg rejection experiments with conspecific eggs) showed that the host strategies of high polymorphism and strong discrimination were equally successful on average at detecting and rejecting their corresponding parasitic gens (figure 3). Thus, interactions with the cuckoo finch have taken these two currently parasitized host species on contrasting evolutionary trajectories, but with similar mean outcomes in terms of natural selection through host rejection behaviour.

The shape of the respective egg rejection frequency distributions (figure 3) may give some insight into selection currently acting on both parties. In tawny-flanked prinias, rejection probability was approximately even for all parasitic-host pairings; such a pattern might be expected if negative frequency-dependent selection on host egg appearance equalizes payoffs for any one parasitic egg phenotype in different host clutches. In red-faced cisticolas, no parasitic eggs had an extremely high probability of being rejected, whereas some had a very high chance of acceptance. We might speculate that the arms race has thus proceeded to a point where a substantial number of parasitic egg phenotypes are able to evade discrimination from certain host phenotypes.

The rattling cisticola, intermediate with respect to both strategies of defence, is not currently parasitized at our study site. Just as several species potentially suitable as common cuckoo hosts show strong rejection behaviour [9], it is likely that the rattling cisticola is a former host that has won the arms race with the cuckoo finch. While some foreign eggs were accepted, experimental eggs were always conspecific and hence likely to be a much closer match to the host clutch than real parasitic eggs; therefore, this does not preclude consistent rejection of cuckoo finch eggs. If the rattling cisticola is indeed a former host, then implementing both strategies appears to have been a highly successful defence against brood parasitism. Ideally, these dual defences would be analysed in a phylogenetic context across a range of hosts to distinguish ancestral from derived levels of defence.

We suggest that both defence strategies are likely to carry costs, however, and are unlikely to be maintained in the absence of an active selection pressure. Such costs may also exacerbate trade-offs inherent to evolving defences. First, while evolving strong egg discrimination may allow a host parent to detect a highly mimetic foreign egg, it may also lead to an elevated risk of rejection errors, causing a host to reject a slightly aberrant egg of its own [6,32]. Depending on how well host signatures have escaped parasitic forgeries, cruder discrimination by certain hosts might reflect an adaptive response to such costs [6] rather than necessarily being constrained by visual performance. Second, high levels of polymorphism may carry costs with respect to other sources of selection on egg appearance, such as camouflage, thermoregulation and protection from solar radiation [16,33]. For example, the degree of polymorphism declined in introduced village weaver *Ploceus cucullatus* host populations released from selection by diederik cuckoos *Chrysococcyx caprius* [33,34], in a direction consistent with protection from solar radiation [33]. Finally, in parasitized populations, we might also speculate that extreme levels of polymorphism could render hosts with certain phenotypes susceptible to other parasitic strains currently adapted to other host species. Such costs may differ among species, potentially influencing the relative contribution of polymorphism and discrimination to a host's eventual balance of defences.

We found that different aspects of egg appearance predicted egg rejection in each host species, strongly suggesting that they used different traits as cues. For example, pattern contrast (total energy) predicted rejection in red-faced cisticolas but not in either of the other species. We have previously shown that those aspects of egg appearance predicting rejection in tawny-flanked prinias were also those that differed most consistently between host and parasitic eggs, and should therefore serve as the most reliable signals of egg identity [18]. The data presented here for an additional currently exploited host species, the red-faced cisticola, generally corroborate this finding: the two traits whose interaction was a significant predictor of rejection (electronic supplementary material, table S3) differed on average between host and real parasitic eggs (electronic supplementary material, table S2). However, we could not detect (albeit with a smaller sample size than for prinias) an effect of the other three traits that also differed significantly between hosts and parasites (electronic supplementary material, table S2), and hence might also function as relatively reliable cues. Our results do clearly suggest that particular traits are unlikely to be intrinsically superior as cues of egg identity (cf. [20]). Rather, they suggest that the information value of different traits is likely to differ among hosts depending on the degree of mimicry for each trait attained by their specialist parasites, and that these should be and are favoured as cues.

A fundamental question in sensory ecology is what information different components of a signal contain, and how that information is used and processed by the receiver in order to make a decision [35]. In general, little work has tested the relative importance of different components of the same signal in animal decision-making. This is the first brood parasitic system for which (i) egg rejection behaviour has been quantified in terms of multiple egg pattern and colour traits, as relevant to the hosts' visual systems (see also [18]), and (ii) intraspecific egg variation has been quantified across multiple traits and hosts. This has revealed that multiple colour and pattern traits are involved in generating phenotypic polymorphisms, and are under selection through host rejection behaviour. The lack of correlation among these numerous different aspects of colour and pattern is unusual [36], and potentially important as it suggests that selection has generated egg phenotypes that maximize information content about egg identity [37]. An integrated approach to studying both the spatial and colour (‘spatiochromatic’) components of a visual signal shows promise for shedding light on the information contained in such signals, their role in behavioural tasks and their evolution.

## References

[1] Davies N. B. 2000 Cuckoos, cowbirds and other cheats. London, UK: T & AD Poyser

[2] Brooke M. de L. Davies N. B. 1988 Egg mimicry by cuckoos *Cuculus canorus* in relation to discrimination by hosts. Nature 335, 630–632 10.1038/335630a0 (doi:10.1038/335630a0)

[3] Kilner R. M. Noble D. G. Davies N. B. 1999 Signals of need in parent–offspring communication and their exploitation by the common cuckoo. Nature 397, 667–672 10.1038/17746 (doi:10.1038/17746)

[4] Welbergen J. A. Davies N. B. 2009 Strategic variation in mobbing as a front line of defence against brood parasitism. Curr. Biol. 19, 235–240 10.1016/j.cub.2008.12.041 (doi:10.1016/j.cub.2008.12.041) 19185495

[5] Soler J. J. Soler M. 2000 Brood-parasite interactions between great spotted cuckoos and magpies: a model system for studying coevolutionary relationships. Oecologia 125, 309–320 10.1007/s004420000487 (doi:10.1007/s004420000487) 28547325

[6] Rothstein S. I. 1982 Mechanisms of avian egg recognition: which egg parameters elicit responses by rejecter species? Behav. Ecol. Sociobiol. 11, 229–239 10.1007/BF00299299 (doi:10.1007/BF00299299)

[7] Langmore N. E. Hunt S. Kilner R. M. 2003 Escalation of a coevolutionary arms race through host rejection of brood parasitic young. Nature 422, 157–160 10.1038/nature01460 (doi:10.1038/nature01460) 12634784

[8] Lahti D. C. 2006 Persistence of egg recognition in the absence of cuckoo brood parasitism: pattern and mechanism. Evolution 60, 157–168 16568640

[9] Davies N. B. Brooke M. L. 1989 An experimental study of co-evolution between the cuckoo, *Cuculus canorus*, and its hosts. II. Host egg markings, chick discrimination and general discussion. J. Anim. Ecol. 58, 225–236 10.2307/4996 (doi:10.2307/4996)

[10] Stoddard M. C. Stevens M. 2010 Pattern mimicry of host eggs by the common cuckoo, as seen through a bird's eye. Proc. R. Soc. B 277, 1387–1393 10.1098/rspb.2009.2018 (doi:10.1098/rspb.2009.2018) PMC2871939 20053650

[11] Takasu F. 2003 Co-evolutionary dynamics of egg appearance in avian brood parasitism. Evol. Ecol. Res. 5, 345–362

[12] Takasu F. 2005 A theoretical consideration on co-evolutionary interactions between avian brood parasites and their hosts. Ornithol. Sci. 4, 65–67 10.2326/osj.4.65 (doi:10.2326/osj.4.65)

[13] Øien I. J. Moksnes A. Røskaft E. 1995 Evolution of variation in egg color and marking pattern in European passerines: adaptations in a coevolutionary arms race with the cuckoo *Cuculus canorus*. Behav. Ecol. 6, 166–174 10.1093/beheco/6.2.166 (doi:10.1093/beheco/6.2.166)

[14] Soler J. J. Møller A. P. 1996 A comparative analysis of the evolution of variation in appearance of eggs of European passerines in relation to brood parasitism. Behav. Ecol. 7, 89–94 10.1093/beheco/7.1.89 (doi:10.1093/beheco/7.1.89)

[15] Gibbs H. L. Sorenson M. D. Marchetti K. Brooke M. de L. Davies N. B. Nakamura H. 2000 Genetic evidence for female host-specific races of the common cuckoo. Nature 407, 183–186 10.1038/35025058 (doi:10.1038/35025058) 11001055

[16] Kilner R. M. 2006 The evolution of egg colour and patterning in birds. Biol. Rev. 81, 383–406 10.1017/S1464793106007044 (doi:10.1017/S1464793106007044) 16740199

[17] Bond A. B. Kamil A. C. 2002 Visual predators select for crypticity and polymorphism in virtual prey. Nature 415, 609–613 10.1038/415609a (doi:10.1038/415609a) 11832937

[18] Spottiswoode C. N. Stevens M. 2010 Visual modeling shows that avian host parents use multiple visual cues in rejecting parasitic eggs. Proc. Natl Acad. Sci. USA 107, 8672–8676 10.1073/pnas.0910486107 (doi:10.1073/pnas.0910486107) 20421497 PMC2889299

[19] Sorenson M. D. Payne R. B. 2001 A single, ancient origin of obligate brood parasitism in African finches: implications for host–parasite coevolution. Evolution 55, 2550–2567 10.1111/j.0014-3820.2001.tb00768.x (doi:10.1111/j.0014-3820.2001.tb00768.x) 11831669

[20] Polačiková L. Grim T. 2010 Blunt egg pole holds cues for alien egg discrimination: experimental evidence. J. Avian Biol. 41, 111–116 10.1111/j.1600-048X.2010.04983.x (doi:10.1111/j.1600-048X.2010.04983.x)

[21] Hockey P. A. R. Dean W. R. J. Ryan P. G. (eds) 2005 Roberts' birds of Southern Africa, VIIth edn Cape Town, South Africa: The Trustees of the John Voelcker Bird Book Fund

[22] Vernon C. J. 1964 The breeding of the Cuckoo Weaver *Anomalospiza imberbis* in southern Rhodesia. Ostrich 35, 260–263 10.1080/00306525.1964.9639425 (doi:10.1080/00306525.1964.9639425)

[23] Endler J. A. Mielke P. W. J. 2005 Comparing color patterns as birds see them. Biol. J. Linn. Soc. 86, 405–431 10.1111/j.1095-8312.2005.00540.x (doi:10.1111/j.1095-8312.2005.00540.x)

[24] Hart N. S. Partridge J. C. Cuthill I. C. Bennett A. T. D. 2000 Visual pigments, oil droplets, ocular media and cone photoreceptor distribution in two species of passerine: the blue tit (*Parus caeruleus* L.) and the blackbird (*Turdus merula* L.). J. Comp. Physiol. A 186, 375–387 10.1007/s003590050437 (doi:10.1007/s003590050437) 10798725

[25] Osorio D. Vorobyev M. 2005 Photoreceptor spectral sensitivities in terrestrial animals: adaptations for luminance and colour vision. Proc. R. Soc. B 272, 1745–1752 10.1098/rspb.2005.3156 (doi:10.1098/rspb.2005.3156) PMC1559864 16096084

[26] Komdeur J. Oorebeek M. van Overveld T. Cuthill I. C. 2005 Mutual ornamentation, age, and reproductive performance in the European starling. Behav. Ecol. 16, 805–817 10.1093/beheco/ari059 (doi:10.1093/beheco/ari059)

[27] Hanlon R. T. Chiao C.-C. Mäthger L. M. Barbosa A. Buresch K. C. Chubb C. 2009 Cephalopod dynamic camouflage: bridging the continuum between background matching and disruptive coloration. Phil. Trans. R. Soc. B 364, 429–437 10.1098/rstb.2008.0270 (doi:10.1098/rstb.2008.0270) 19008200 PMC2674088

[28] Stevens M. Párraga A. Cuthill I. C. Partridge J. C. Troscianko T. 2007 Using digital photography to study animal coloration. Biol. J. Linn. Soc. 90, 211–237 10.1111/j.1095-8312.2007.00725.x (doi:10.1111/j.1095-8312.2007.00725.x)

[29] R Development Core Team. 2010 R: a language and environment for statistical computing. Vienna, Austria: R Foundation for Statistical Computing

[30] Ruxton G. D. 2006 The unequal variance *t-*test is an underused alternative to Student's *t*-test and the Mann–Whitney *U*-test. Behav. Ecol. 17, 688–690 10.1093/beheco/ark016 (doi:10.1093/beheco/ark016)

[31] Grueber C. E. Nakagawa S. Laws R. J. Jamieson I. G. 2011 Multimodel inference in ecology and evolution: challenges and solutions. J. Evol. Biol. 24, 699–711 10.1111/j.1420-9101.2010.02210.x (doi:10.1111/j.1420-9101.2010.02210.x) 21272107

[32] Davies N. B. Brooke M. de L. Kacelnik A. 1996 Recognition errors and probability of parasitism determine whether reed warblers should accept or reject mimetic cuckoo eggs. Proc. R. Soc. Lond. B 263, 925–931 10.1098/rspb.1996.0137 (doi:10.1098/rspb.1996.0137)

[33] Lahti D. C. 2008 Population differentiation and rapid evolution of egg color in accordance with solar radiation. Auk 125, 796–802 10.1525/auk.2008.07033 (doi:10.1525/auk.2008.07033)

[34] Lahti D. C. 2005 Evolution of bird eggs in the absence of cuckoo parasitism. Proc. Natl Acad. Sci. USA 102, 18 057–18 062 10.1073/pnas.0508930102 (doi:10.1073/pnas.0508930102) PMC1312405 16326805

[35] Stevens M. 2010 Sensory ecology, evolution, and behavior. Curr. Zool. 56, i–iii

[36] McKinnon J. S. Pierotti M. E. R. 2010 Colour polymorphism and correlated characters: genetic mechanisms and evolution. Mol. Ecol. 19, 5101–5125 10.1111/j.1365-294X.2010.04846.x (doi:10.1111/j.1365-294X.2010.04846.x) 21040047

[37] Dale J. Lank D. B. Reeve H. K. 2001 Signaling individual identity versus quality: a model and case studies with ruffs, queleas, and house finches. Am. Nat. 158, 75–86 10.1086/320861 (doi:10.1086/320861) 18707316

